# The Effect of Cellular Coenzyme Q_10_ Deficiency on Lysosomal Acidification

**DOI:** 10.3390/jcm9061923

**Published:** 2020-06-19

**Authors:** Robert A. Heaton, Simon Heales, Khalid Rahman, Darren W. Sexton, Iain Hargreaves

**Affiliations:** 1 School of Pharmacy, Liverpool John Moore University, Byrom Street, Liverpool L3 3AF, UK; k.rahman@ljmu.ac.uk (K.R.); D.W.Sexton@ljmu.ac.uk (D.W.S.); I.P.Hargreaves@ljmu.ac.uk (I.H.); 2 Neurometabolic Unit, National Hospital, Queen Square, London WC1N 3BG, UK; Simon.Heales@gosh.nhs.uk; 3 Enzyme Unit, Chemical Pathology, NIHR BRC Great Ormond Street Hospital, Foundation Trust, London WC1N 3JH, UK; 4 NIHR BRC and UCL Great Ormond Street Institute of Child Health, London WC1N 1EH, UK

**Keywords:** coenzyme Q_10_, lysosome, mitochondrial, organelles, competitive inhibition, lysosomal pH

## Abstract

Coenzyme Q_10_ (CoQ_10_) deficiency currently represents the only treatable mitochondrial disorder, however, little is known about how it may affect other organelles. The lysosome has been found to have a large concentration of CoQ_10_ localised at its membrane; additionally, it has been suggested that it plays a role in the normal acidification of the lysosomal lumen. As a result, in this study we assessed the effect of CoQ_10_ deficiency on lysosomal acidification. In order to investigate this, a neuronal cell model of CoQ_10_ deficiency was established via the treatment of SH-SY5Y cells with para-aminobenzoic acid (PABA). This method works through the competitive inhibition of the CoQ_10_ biosynthetic pathway enzyme, CoQ_2_. A single 1 mM (5 days) treatment with PABA resulted in a decrease of up to 58% in cellular CoQ_10_ (*p* < 0.05). It was found that this resulted in a significant decrease in fluorescence of both the LysoSensor (23%) and LysoTracker (35%) probes used to measure lysosomal pH (*p* < 0.05). It was found that subsequent treatment with CoQ_10_ (5 µM, 3 days) was able to restore cellular CoQ_10_ concentration (*p* < 0.005), which was associated with an increase in fluorescence from both probes to around 90% of controls (*p* < 0.05), suggesting a restoration of lysosomal pH. This study provides insights into the association between lysosomal pH and cellular CoQ_10_ status and the possibility that a deficit in the status of this isoprenoid may result in an impairment of lysosomal acidification.

## 1. Introduction

Coenzyme Q_10_ (CoQ_10_) is a lipid-soluble vitamin-like molecule synthesised in the mitochondria where it plays a vital role in the mitochondrial electron transport chain (METC), passing electrons derived from both complex I (NADH ubiquinone oxidoreductase; EC 1.6.5.3) and II (succinate ubiquinone reductase; EC 1.3.5.1) to complex III (ubiquinol cytochrome c reductase; EC 1.10.2.2) allowing a continuous passage of electrons within the chain which is required for the process of oxidative phosphorylation and the concomitant production of ATP [1]. Additionally, CoQ_10_ functions as an important lipid soluble antioxidant within cellular membranes and lipoproteins, protecting them from free radical-induced oxidative damage [2].

**Figure 1:**
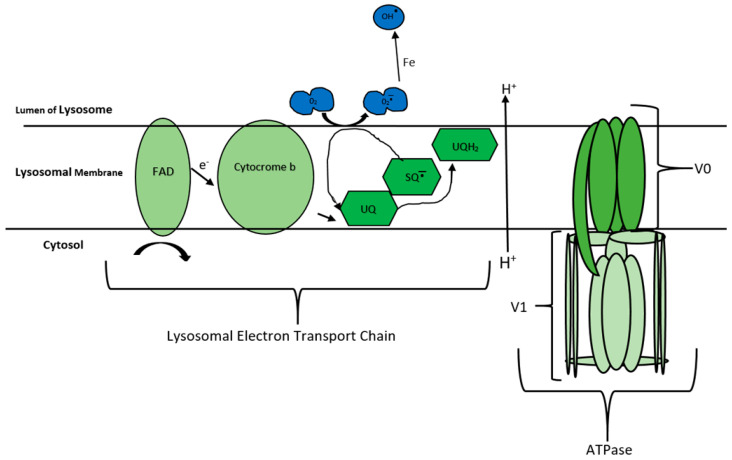
The lysosomal electron transport chain (LETC), adapted from Gille and Nohl [14]. The figure indicates how coenzyme Q_10_ (CoQ_10_) oxidation and reduction are utilised by the lysosome to move H^+^ into the lumen. As eluded to by Gille and Nohl [14]. The oxidation of cytoplasmic nicotinamide adenine dinucleotide (NAD) to NADH^+^ by the flavin-adeine dinucleotide (FAD) present in the lysosomal membrane, leads to the single electron transfer to cytochrome b. Additionally, it has been reported that the oxidation of cytochrome b was found to be proportional to the amount of ubiquinone (UQ) present in the lysosomal membrane. Ubiquinone structure allows for the transfer of two electrons leading to the production of either partially reduced ubisemiqinone or the fully reduced ubiquinol. CoQ_10_’s structure then allows it to pass freely into the lysosomal lumen, transferring the electrons via molecular oxygen. The basic structure of the lysosomal ATPase is also outlined and is adapted from the paper by Zoncu, et al. [15]. The structure and function of this ATPase is similar to that found in the mitochondria, although it is involved in the hydrolysis of ATP rather than the synthesis of this molecule. It consists of 13 subunits and is divided into 2 domains, V0 and V1. The V0 domain is involved in translocating protons (H^+^)’s across the lysosomal membrane and into the lumen. The V1 domain is involved in the hydrolysis of ATP which provides the energy for the translocation of H^+^ ions against the H^+^ concentration gradient for transport into the lumen. [15,16]. Abbreviations: FAD, flavin-adeine dinucleotide; UQ, ubiquinone; SQ, semiquinone.

A primary CoQ_10_ deficiency, which results from a genetic defect in the CoQ_10_ biosynthetic pathway, currently represents the only treatable METC disorder, and can have quite a heterogeneous clinical presentation which can be divided into five major phenotypes: encephalomyopathy, severe infantile multisystemic disease, nephropathy, cerebellar ataxia, and isolated myopathy [3]. Lowered tissue levels of CoQ_10_ have also been reported as a secondary consequence of disease pathophysiology, as well as drug therapy, and are known as secondary CoQ_10_ deficiencies [4]. CoQ_10_ supplementation has also shown some therapeutic potential in the treatment of a number of diseases, including mitochondrial disease [5], cardiovascular disease [6,7,8] and endothelial dysfunction [9]. The clinical benefit elicited by CoQ_10_ in the treatment of these disorders possibly relies on the ability of Q_10_ to restore electron flow in the METC as well as its cellular antioxidant capacity [10]. In this context, the preponderance of current studies assessing CoQ_10_’s therapeutic value have focused on its effects in the METC and, thus, on diseases linked to mitochondrial dysfunction [11,12,13]. However, in addition to its important electron carrier role in the METC, CoQ10 has also been reported to be present in the lysosomal membrane in high concentrations [2,14], where it is has been suggested to play an important role in the tentative electron transport chain of this organelle (lysosomal electron transport chain (LETC)) [14] (Figure 1).

The lysosome can be considered to be a vital organelle for cell survival, in that it has an active role in a multitude of cellular homeostatic pathways [17]. They are membrane bound organelles responsible, via enzymatic digestion, for the recycling of complex molecules and organelles such as mitochondria [18,19,20]. Lysosomes contain over 70 different hydrolytic enzymes that break down and digest a plethora of different cellular products such as proteins, DNA, RNA, polysaccharides and lipids [21,22]. Changes in lysosomal function have been linked to over 30 different diseases known as lysosomal storage diseases (LSD) [23].

The majority of lysosomal enzymes are acidic hydrolyses requiring an acidic environment for optimum activity. Thus, the lysosomal lumen pH is intrinsically linked to its functionality [24,25]. When compared to the relatively neutral pH of the cytosol (7.2), it is clear that the lysosome must have a mechanism for the movement of H^+^ ions into the lumen against the concentration gradient. The lysosomal electron transport chain, LETC, was first observed by Gille and Nohl [14]. This is not dissimilar to the electron transport chain found in the mitochondria (METC) and could explain the presence of CoQ_10_ in the lysosomal membrane (Figure 1) [26,27,28,29,30]. As shown in Figure 1, CoQ_10_ is utilised in the LETC for its oxidation/reduction abilities. Gille and Nohl [14] noted that electrons are passed from Cytocrome b, onto ubiquinone (UQ), reducing to ubisemiquinone (SQ^−^), then an O_2_ molecule inside the lumen acts as a final acceptor, oxidising SQ^−^ and moving H^+^ ions into the lysosome. Thus, it is not unreasonable to assume that a CoQ_10_ deficiency may result in an impairment of lysosomal activity as the result of a deacidification of the organelle. However, as yet, it is uncertain whether CoQ10 plays a fundamental role in maintaining lysosomal pH and this requires confirmation.

In view of the multi-organ presentation of lysosomal disorders [31], lysosomal dysfunction may be an important factor to consider in diseases associated with a CoQ_10_ deficiency. At present, however, no studies have assessed the effect of a CoQ_10_ deficiency on lysosomal acidification which may have important clinical and therapeutic consequences. Therefore, the aim of the present study was to assess the effect of a pharmacologically-induced CoQ_10_ deficiency on lysosomal acidification and, subsequently, whether CoQ_10_ supplementation can be used to reverse this effect. The results of this study will provide important information about the role of CoQ10 in maintaining lysosomal acidification.

## 2. Materials and Methods

### 2.1. Cell Culture

All experiments were carried out using the SH-SY5Y neuroblastoma cell line provided by the UCL Great Ormond Street of Child Health, London. The effect of CoQ_10_ deficiency was of interest in these cells specifically in light of CoQ_10_’s link to neurological disorders [32]. Prior to treatments, all cells were grown in Dulbecco’s Modified Eagle’s medium–high glucose in T75 cell culture flasks. Cells were passaged at 70–80% confluent, with passage number kept within 18–25 in order to obtain reproducible results, as per standard set out by previous studies [33]. All fluorescent based assays were carried out in black flat bottomed 96-well plates, unless otherwise stated. Additionally, all cells grown for CoQ_10_ analysis were cultured in T75 cell culture flasks. All reagents were purchased from Sigma-Aldrich, unless stated. Both the LysoSensor and LysoTracker were purchased from Thermo Fisher scientific.

Cells were treated with a single dose of 1 mM para-aminobenzoic acid (PABA) for 5 days following the methods previously outlined by Duberley, et al. [34]. Once seeded in wells or T75 cells were allowed to rest for 10 min before the addition of PABA media. The 1 mM PABA media was made on the day and kept at 37 °C for at least 20 min before application to the cells. PABA works through the competitive inhibition of CoQ_10_ biosynthesis and competes with para-hydroxybenzonate for the active site of polyprenyl-4-hydroxybenzoate transferase [35].

### 2.2. CoQ_10_ Quantification

The CoQ_10_ concentration of all neuronal cell samples in this study was determined using reverse-phase HPLC with UV detection at 275 nm, following the method previously described by Duncan, et al. [36]. A hexane: Ethanol (5:2) solution was used to extract the CoQ_10_ from the cells, following the method previously described by Duberley, Abramov, Chalasani, Heales, Rahman and Hargreaves [34]. Once extracted, CoQ_10_ was resuspended in HPLC grade ethanol, and separation and quantification was achieved using a reverse phase c18 column (Phenomenex, Torrance, CA, USA) using a mobile phase comprised of methanol, ethanol and perchloric acid (700:300:1.2) at a flow rate of 0.7 mL/min. CoQ_10_ detection was achieved using a Agilent 1200 series UV detector at 275 nm and its concentration was calculated through comparison with standard CoQ_10_ solution.

### 2.3. Total Protein Analysis

Cellular protein concentration was determined using the detergent compatible protein assay (DC protein assay) kit available from BIO RAD which is a modified version of the Lowry, et al. [37]. The cellular protein concentration was standardised against bovine serum (BSA) standards 0 to 200 μg/mL. Cell samples and BSA standards (200 μL) in Eppendorf tubes (1.5 mL) were treated with protein assay reagent A (100 mL) and protein assay reagent B (800 mL), vortex-mixed and incubated at room temperature for 25 min. Following the incubation period, the absorbance of these samples was measured at 750 nm using a spectrophotometer (NorthStar Scientific, Leeds, UK, Uvikon xs).

### 2.4. CoQ_10_ Treatment

Following PABA treatments, cells were treated with CoQ_10_, in order to evaluate the effect of the restoration of CoQ_10_. CoQ_10_ was solubilised in ethanol and then incubated with media at 37 °C for 15 min prior to addition to the SH-SY5Y cells. The concentration of CoQ_10_ in the media was confirmed by HPLC analysis [36]. On the day of experiment a 5 µM CoQ_10_ solution was made up in media and kept at 37 °C for 20 min. Cells were treated with a 5 µM CoQ_10_ since this concentration of CoQ_10_ has been reported to elicit some biochemical efficacy in CoQ_10_-deficient fibroblasts in the study by Lopez et al. (2010) [29]; increasing cellular ATP status and ameliorating oxidative stress [38]. PABA media was removed and cells washed with 1X PBS, CoQ_10_ (5 µM) treatment media with fresh PABA (1 mM) was then added (200 µL 96-well plate, 10 mL T75 culture flask) and cells incubated for 3 days according to the method of Duberley, et al. [39]. Control cells were incubated for 8 days in culture with the media being refreshed after 5 days [30].

### 2.5. Lysosomal pH Measurements

For the determination of lysosomal pH both LysoSensor blue DND-167 and LysoTracker red DND-99 were used. Both of these probes have been developed specifically to assess lysosomal pH [40].

### 2.6. LysoTracker

The LysoTracker (LT) probe is made up of a fluorophore linked to a weak base that is only partially protonated at a neutral pH, thus, it can be used to measure acidic compartments such as the lysosome [41,42,43]. On the day of experiment a LT media was made with a final concentration of 1 µM in normal cell culture media prior to staining and kept at 37 °C in the dark until use. After PABA treatment was complete, media was removed, and cells washed twice with 1X PBS. The LT media was then added (200 µL) and cells incubated for 20 min. Once incubated the staining media was removed and cells were washed with PBS as before. Cells were then lifted from wells using trypsin (20 µL, 5 min). Once lifted, fresh media was added to inhibit the trypsin, and cells taken to the flow cytometer for measurement [44].

Flowcytometry was performed on an Accuri™ C6 flow cytometer (BD Biosciences, San Jose, CA, USA) with a 488 nm laser; FL1 was 533/30 nm, FL2 585/40 nm and FL3 670 nm for detection of fluorescence. The forward scatter (FSC), side scatter (SSC) and fluorescence data were obtained with logarithmic scale configuration, gating was performed as shown in supplemental data under Figure S4.

### 2.7. LysoSensor

On the day of measurements, LysoSensor (LS) staining media was made with standard culture media, to a final solution of 1 µM; this media was then kept in the dark at 37 °C until staining. As with LT, the cell media was removed, and cells washed. LS media was then added (200 µL) and cells incubated for 1 h. Following this incubation, the media was then removed, and cells washed twice with 1X PBS. Cells were then left in 100 µL of fresh PBS and fluorescence measured quickly. All LS measurements were made on a Clariostar plate reader with excitation at wavelength 373 nm and emission detection at 425 nm, following the method described by Ma, et al. [45], Anway, et al. [46], Altan, et al. [47], Papakrivos, et al. [48].

### 2.8. PH Calibration

pH calibrants were purchased from Sigma Aldrich, clear calibrants were used and each calibrant’s background fluorescence was analysed before experiments; pH of 4.5, 5.1, 6, and 7 were used as this was deemed to be within normal physiological conditions for the lysosome [49].

For each experiment, a pH calibration curve was created as per the LS method. Cells were incubated (37 °C) in 96-well plates in normal cell culture medium for 5 days before pH calibration. The media was removed, and cells washed with 1XPBS. Cells were then stained by adding 200 µL of LS staining media (1 µM LS in pre-warmed media as described above) and incubating for 1 h (37 °C). The staining media was then removed, and cells were washed twice with 1X PBS. This was followed by the addition of 100 µL of the selected pH calibrant and the cells were incubated for a further 10 min; the fluorescence intensity was measured using a plate reader, as outlined by Ma, Ouyang, Werthmann, Thompson and Morrow [45].

### 2.9. Imaging Probes

Assessment of the uptake of the probes was carried out using the Leica DMI6000 fluorescent microscope, with the Leica LAS X software. Cells were grown on clear 6-well plates and seeded at 80,000 cell/mL. PABA treatments were carried out as described above with all media volumes set at 2 mL. After treatments cells were twice washed with 1X PBS and stained with LT and LS probes, as outlined above. Following the addition of fresh media (2 mL), the cells were then subjected to imaging using fluorescence microscopy.

### 2.10. Statistical Analysis

All results are expressed as mean ± standard error of the mean (SEM), error bars represent SEM. Data was subjected to Normality testing and once appropriate parametric analyses were confirmed, ANOVAs (groups ≥ 3) with Tukey’s post-hoc test or Paired T tests as indicated, were used to compare data sets. An alpha value of 0.05 was considered significant. The ‘*n*’ number represents the number of individual observations. The groups compared by statistical analysis are shown in the figure legends of the results section of the manuscript. All statistical analyses were carried out using R-Studio (Version 1.2.5033). ImageJ software was used to visualise images of the probes.

## 3. Results

### 3.1. PABA Treatment

**Figure 2:**
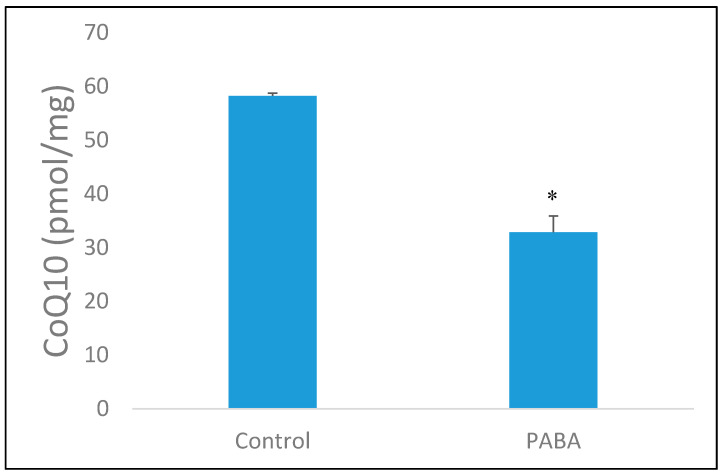
The endogenous CoQ_10_ concentration in both control and para-aminobenzoic acid (PABA)-treated (1 mM, 5 day) SH-SY5Y cells. CoQ_10_ concentration standardised to protein baseline (mg of protein). Data available in supplemental data under Table S2. Levels of significance * *p* < 0.05; *n* = 10.

Analysis showed that cellular CoQ_10_ concentration was significantly decreased (*p* < 0.05) in PABA-treated cells when compared to control cells. A 5-day treatment with 1 mM PABA induced a 58% decrease in cellular CoQ_10_ content (Figure 2). It was found that treatment of cells with higher concentrations of PABA did not result in a further deficiency in CoQ_10_ (results not shown), thus, 1 mM PABA was used throughout this study. The effect of PABA on cell viability was also assessed and was found to have no significant effect (data not shown), this is in agreement with a previous study undertaken by Duberley, Abramov, Chalasani, Heales, Rahman and Hargreaves [34].

### 3.2. CoQ_10_ Treatment

Following PABA treatment for 5 days, media was removed, and cells incubated in media containing 5 µM CoQ_10_ and fresh PABA (1 mM). This concentration of CoQ_10_ was selected since it has been reported by Lopez et al. (2010) [29] to increase cellular ATP status and ameliorate oxidative stress in CoQ_10_-deficient fibroblasts.

**Figure 3:**
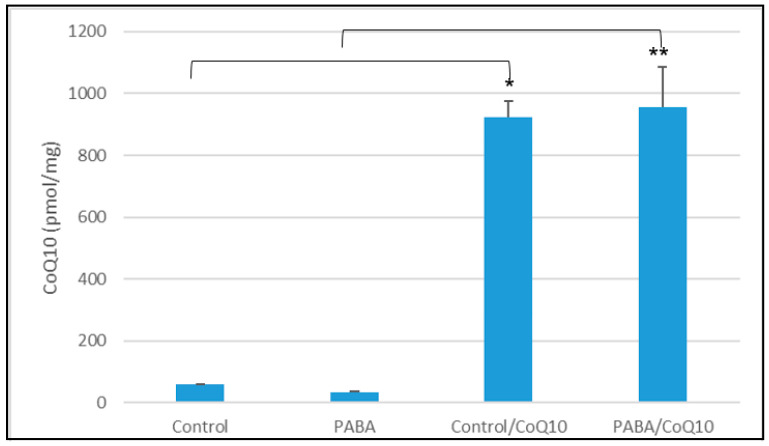
The cellular CoQ_10_ concentration, after treatment with 5 µM CoQ_10_ over 3 days. Figure shows concentration of CoQ_10_ after PABA treatment and subsequent CoQ_10_ treatment. CoQ_10_ concentration standardised to protein baseline (mg of protein). Comparison of groups was carried out as follows: control group vs. PABA, Control group vs. Control CoQ_10_ treated, PABA group vs. PABA CoQ_10_ treated. Data available in supplemental data under Table S3. Level of significance * *p* < 0.05, ** *p* < 0.05; *n* = 26.

The CoQ_10_ status of PABA-treated cells was found to be significantly increased when compared to control cells, after a three-day incubation with 5 µM CoQ_10_ (*p* < 0.005) (Figure 3). This result was consistent with a previous study by Duberley, Heales, Abramov, Chalasani, Land, Rahman and Hargreaves [39] where treatment resulted in an average 25-fold increase in cellular CoQ_10_ status (compared to CoQ_10_-deficient cells).

### 3.3. LysoTracker

**Figure 4:**
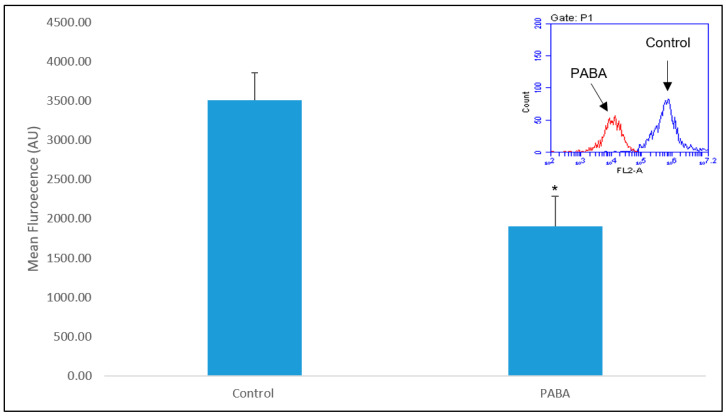
Shows the mean LysoTracker DND-99 fluorescence of PABA-treated cells vs. controls. Additionally, this figure shows the fluorescence shift after PABA treatment. Data available in supplemental data under Figure S4. Level of significance * *p* < 0.05; *n* = 26.

Analysis showed that there was a significant difference (*p* < 0.005) between the fluorescence of the non-PABA-treated cells and the CoQ_10_-deficient (PABA-treated) cells (Figure 4). In addition, there was a 35% (*p* < 0.05) decrease in median fluorescence on average in CoQ_10_-deficient cells when compared to the control (Figure 4).

An assessment of whether PABA interferes with the LT assay was also undertaken, this was to ensure that PABA was not affecting the results. It was found that when PABA and LT were tested simultaneously no effect on the florescence of LT was evident.

### 3.4. LysoSensor

**Figure 5:**
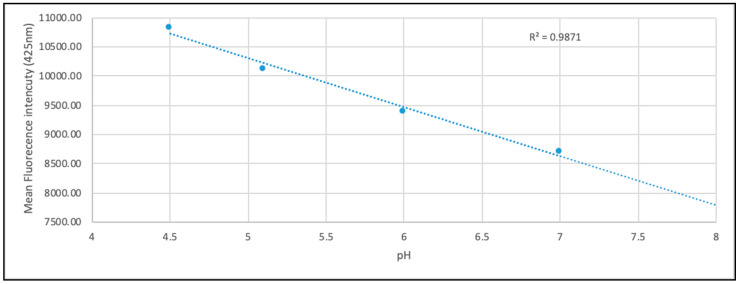
Standard pH curve from LysoSensor DND-167 after treatment of cells with standard pH solutions. Represents the mean fluorescence of all pH calibration data. *n* = 8 (for each calibrant). Data available in supplemental data under Table S5.

**Figure 6:**
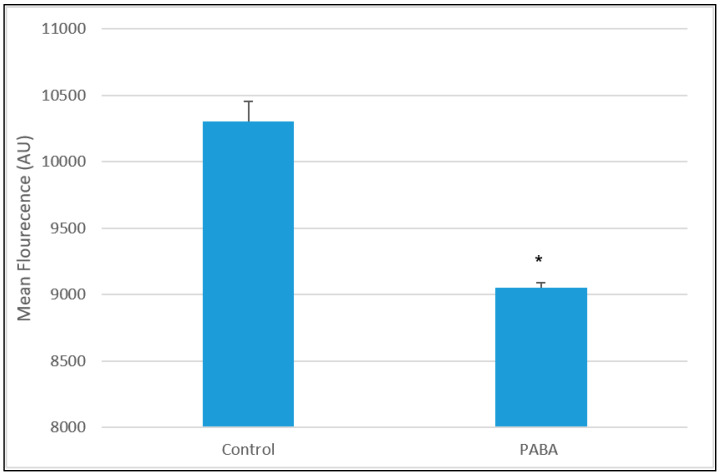
Mean LysoSensor fluorescence for PABA-treated cells vs. controls. Data available in supplemental data under Table S6. Significance levels * *p* < 0.05; *n* = 94.

To assess the lysosomal pH, a calibration curve was constructed to allow quantification of pH, prior to experiments (Figure 5); fluorescence of pH calibrants was measured and found to be within the same range as the PBS used in experiments. Treatment with PABA (1 mM 5 days) showed a significant decrease in LS fluorescence when compared to the control (*p* < 0.05). The results show a decrease of 23% in LS fluorescence (Figure 6).

**Figure 7:**
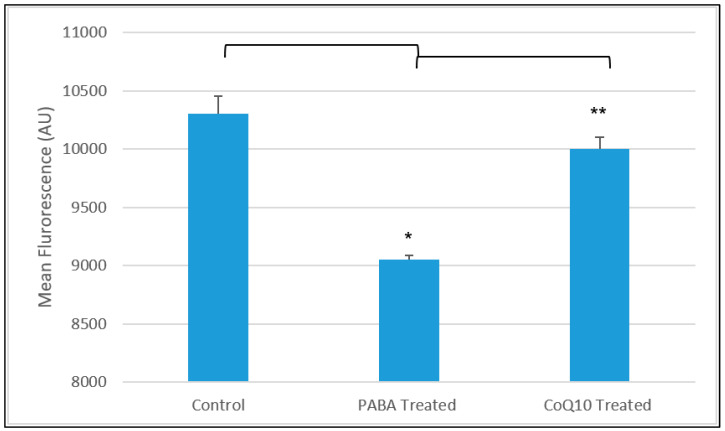
Mean fluorescence of control, PABA-treated cells and CoQ_10_ (5 µM) treated cells in the presence of PABA using data obtained in Figure 6. PABA-treated and CoQ_10_-treated (in the presence of PABA) cells following 3 days of incubation. Control and PABA-treated cells had been cultured for 5 days prior to this experiment as outlined in the cell culture section of the Material and Methods. Following incubation with CoQ_10,_ the LysoSensor (LS) fluorescence is significantly increased when compared to the PABA-treated cells. Comparisons of groups was carried out as follows: Control cells vs. PABA-treated cells, control cells vs. CoQ_10_-treated cells, PABA-treated cells vs. CoQ_10_-treated cells. Data available in supplemental data under Table S6/7. Significance level * *p* < 0.05; *n* = 94, ** *p* < 0.05, *n* = 94.

Following 5 days of treatment with PABA, cells were incubated with CoQ_10_ (5 µM for 3 days) in the presence of fresh PABA (Figure 7). Analysis of this data showed that LS fluorescence was significantly increased when compared to the PABA-treated cells (*p* < 0.05), although it did not exceed 90% of the control value. Using the calibration curve, the pH of the neuronal cell showed a possible increased from 5.1 to 6.2 following PABA-induced diminution of CoQ_10_. The following CoQ_10_ treatment corresponded to a possible decreased in pH of 5.4 according to the pH calibration curve (Figure 5).

### 3.5. Visulisation of Probes

To assess the uptake and localisation of the probes, fluorescent imaging of the cells was carried out. However, images of the LS blue DND-167 could not produce sufficient resolution at X40 (objective); therefore, future analysis of this probe will be required.

**Figure 8:**
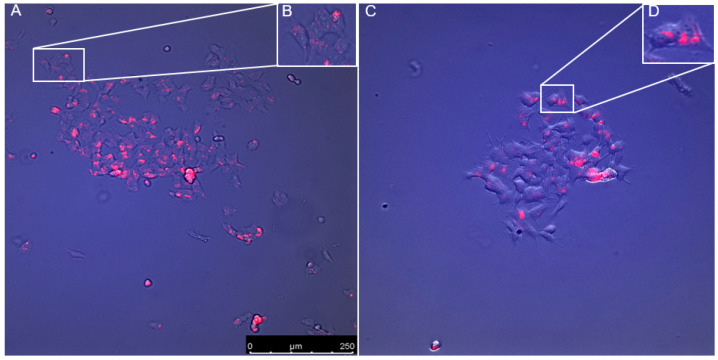
Distribution of the LysoTracker DND-99 probe in both control cells (**A**) and PABA-treated cells (**B**). (**C**) and (**D**) show small localisation of LysoTracker in the cells. Suggests that PABA has little to no effect on the cellular distribution of the probe. Images taken using the Leica DMI6000 fluorescent microscope, with the Leica LAS X software, at x40 (objective).

Analysis of the cellular distribution of the LT probe indicates that it was taken up by the cells and held in small pockets within the cell membrane (Figure 8); this would indicate that the probe was held within acidic organelles. Additionally, this shows that there was minimum fluorescence across the cell outside of these small pockets. Visualisation of control cells and PABA-treated cells suggest that the cellular distribution of the LT probe is unaffected by PABA treatment, however more data is required for confirmation.

## 4. Discussion

The results of this study have shown that a 50% decrease in cellular CoQ_10_ status can be associated with a 20% decrease in lysosomal fluorescence indicating a change in lysosomal pH (Figure 6).

Lysosomal function is imperative to cellular homeostasis, and CoQ_10_ could play a fundamental role in maintaining lysosomal acidification [14]. The effect of cellular CoQ_10_ deficiency on mitochondrial function has been investigated previously in a number of studies reported by Ben-Meir, et al. [50], Shults, et al. [51]. However, to the best of our knowledge the effect of CoQ_10_ deficiency on lysosomal acidification has not been reported.

Following the work of Duberley, Heales, Abramov, Chalasani, Land, Rahman and Hargreaves [39] a CoQ_10_-deficient neuronal cell line was established. Incubating the neuroblastoma-derived SHSY-5Y cells with 1 mM PABA for five days, led to a decrease in cellular CoQ_10_ concentration ranging from 50 to 58% of the normal level. This enabled the analysis of CoQ_10_ deficiency on lysosomal acidification to be undertaken.

This study employed two independent fluorescent probes in order to evaluate lysosomal pH. Firstly, the LS probe was used, incorporating a plate reader method. LS is a fluorophore designed to accumulate within acidic organelles as a result of protonation. Additionally, a result of this protonation is fluorescence quenching of the probe by its weak base side chain resulting in an increased fluorescence intensity [52]. The pK_a_ of LS blue DND-167 is 5.1 meaning the probe is ideal for measuring low pH environments. In this study a set of standard pH controls were set up alongside each experiment, which showed that the change in fluorescence was proportional to the change in pH (Figure 5).

A decrease in cellular CoQ_10_ concentration was found to result in a significant decrease in LS fluorescence intensity when compared to control cells. To ensure that this effect was not due to PABA fluorescence or direct interaction with the probe, a number of controls were run, including empty wells and wells containing only PABA media. No significant difference was found in any control, indicating that the modulated fluorescence effects were due to a decrease in CoQ_10_ status. The results, shown in Figure 6, suggest that a decrease in CoQ_10_ concentration results in a decrease in lysosomal acidification. This study also attempted to create a standard curve for LS fluorescence intensity compared to pH, using pH standard solutions. Combining the results from across all experiments gave a linear relationship between pH and mean fluorescence intensity (Figure 5). However, analysis of individual experiments gave generally non-linear results, suggesting that a more sensitive probe will be required to calculate definitive pH values, as day-to-day variations may compromise the results.

The results from the LT probe also show a significant decrease in fluorescence intensity after PABA treatment, when compared to the control (Figure 4). The LT probe is a member of the same family of pH-sensitive probes as LS and deploys a similar mechanism of action [34]. Weakly-based amines selectively accumulate in low pH environments, where the probe is protonated, increasing its fluorescence. Previous peer-reviewed studies have successfully shown that changes in LT fluorescence can be attributed to large changes in lysosomal pH, though more subtle changes are difficult to ascertain [43]. Where consideration of altered LT fluorescence intensity may be linked to changes in size and/or quantity of lysosome within cells, imaging was attempted to mitigate this and found little to no observable changes in LT distribution (Figure 8). Our sizeable change in LT fluorescence intensity combined with a lack of identifiable changes in LT distribution in our imaging, facilitate the most probable conclusion of change in fluorescence being attributed to change in lysosomal pH. This is also applicable to the use of LS probes [34] and, therefore, further studies will be required to assess the lysosomal content of the neuronal cells before we can confirm or refute that the decrease in fluorescence intensity is specifically caused by a change in lysosomal pH. With this caveat in mind however, the most probable reason for the observed changes in fluorescence, is due to changes in lysosomal pH inflicted by CoQ_10_ concentration changes.

Evaluation of the uptake and localisation of both the LT and LS probes was carried out (Figure 8) using fluorescence microscopy at x40 (objective). However, this did not have sufficient magnification to observe the LS, thus further microscopic studies will be needed to confirm the LS. The analysis of the LT probe showed it was taken up by the cells and localised in small pockets within the cytoplasm (Figure 8). Following data provided by Thermo Fishier and previous studies, it is reasonable to assume that these small pockets of fluorescence within the cell are acidic organelles such as lysosomes.

This study also evaluated the effect of CoQ_10_ supplementation on the CoQ_10_-deficient cell model (Figure 3). This was done to evaluate if the effects of CoQ_10_ deficiency were reversible. Assessment of CoQ_10_ supplementation showed a significant increase in both LS and LT fluorescence intensity when compared to PABA-treated cells (Figure 7). In view of the antioxidant function of CoQ_10,_ a decrease in the concentration of cellular CoQ_10_ will cause an increase in reactive oxygen species (ROS) generation and may result in oxidative stress, as indicated in the study by Duberley, Abramov, Chalasani, Heales, Rahman and Hargreaves [34]. Interestingly, it has been reported that the lysosome itself is susceptible to oxidative stress-induced impairment [53], and, therefore, a deficit in CoQ_10_ status may adversely affect lysosomal acidification by impairing the activity of the LETC and/or causing oxidative damage to the organelle. However, at present it is uncertain what causes lysosomal deacidification resulting from the PABA-induced CoQ_10_ deficiency and this will form the basis of further work. Interestingly, supplementation with 5 μM CoQ_10_ was not able to fully restore lysosomal pH to control levels. In view of the significant increase in cellular CoQ_10_ status (compared to control levels) of the PABA-treated neurons following treatment with 5 µM CoQ_10_ (Figure 3), it is at present uncertain whether the restoration of lysosomal pH may be elicited by more physiological levels of cellular CoQ_10_ that falls within control limits. In this regard, future studies will be undertaken to assess the effect of lower dosage CoQ_10_ treatments on lysosomal pH to determine the cellular level of CoQ_10_ required to restore lysosomal pH in the CoQ_10_-deficient neurons.

It is uncertain at present by what mechanism(s) the deficit in neuronal cell CoQ_10_ status has caused an increase in lysosomal pH, in view of the roles ascribed to CoQ10 in both the LETC [10] and METC [4]. Importantly, the V-ATPase has a major role in maintaining lysosomal pH via its ability to pump protons into the lumen of the organelle [25]. However, the data shown in this study do not detract from our finding that decreased CoQ_10_ availability leads to a perturbation of lysosomal homeostasis. Furthermore, a diminution of CoQ_10_ in the lysosomal membrane may also impair the proton pumping capability of the LETC [4] which may also impact upon lysosomal pH. Although the possibility arises that both of these mechanisms may be the cause of the deacidification of the lysosome following a deficit in CoQ_10_ status, further studies will be undertaken to confirm or refute this possibility.

**Figure 9:**
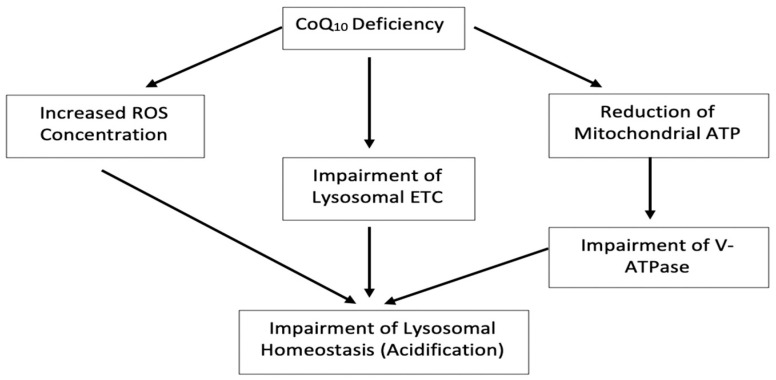
Tentative schematic of possible mechanisms of how CoQ_10_ deficiency may influence the acidification of lysosomes.

In conclusion, the results from the present study indicate that a CoQ_10_ deficiency results in impairment of lysosomal acidification. It would be reasonable to assume that this change in pH would lead to a decrease in lysosomal function. This study also found that treatment with exogenous CoQ_10_ (5 µM) restored the fluorescence to an average of 90% of the controls, suggesting a restoration of lysosomal pH. This suggest that a deficit in cellular CoQ_10_ status may impact upon on lysosomal function as well as its widely described effect on the mitochondria. The possible mechanisms by which CoQ_10_ exerts its effects are shown in Figure 9, however, further work is required to elucidate the relationship between cellular CoQ_10_ status and lysosomal function.

## Supplementary Materials

The following are available online at https://www.mdpi.com/2077-0383/9/6/1923/s1, Table S2: Cellular CoQ_10_ concentration pre and post PABA treatment, Table S3: Cellular CoQ10 concentration post CoQ10 supplementation, Table S4: Cellular CoQ_10_ concentration post CoQ_10_ supplementation, Figure S4: Flow cytometry analysis of cellular LS fluorescence pre and post PABA treatment, Table S5: Mean LS fluorescence after standard pH solution exposure, Table S6/7: analysis of LS florescence during CoQ_10_ deficiency and supplementation.
