# Supplementary material for: The Effect of Cellular Coenzyme Q10 Deficiency on Lysosomal Acidification
Source: J Clin Med. 2020 Jun 19;9(6):1923. doi: 10.3390/jcm9061923 (PMC7355799; doi:10.3390/jcm9061923)
Supplement: Supplementary file 1 [file jcm-09-01923-s001.pdf]

Supplemental Data

Table S2

Cellular CoQ_{10} concentration pre and post PABA treatment

Control Cells CoQ10 pmol/mg	PABA treated cells CoQ10 pmol/mg
58.5	30.0
56.5	29.0
57.5	31.5
60.0	32.0
58.0	54.0
60.0	31.5
61.0	29.5
57.0	30.0
55.0	34.0
59.0	27.0

	Control Cell CoQ10 pmol/mg	PABA Cell CoQ10 pmol/mg
**Mean**	58.25	32.85
**Standard Deviation**	1.83	7.67
**SEM**	0.58	2.43

t-Test: Two-Sample Assuming Equal Variances

	Variable 1	*Variable 2*
Mean	58.25	32.85
Variance	3.34722222	58.8361111
Observations	10	10
Pooled Variance	31.0916667	
Hypothesized Mean Difference	0	
df	18	
t Stat	10.1858369	
P(T<=t) one-tail	3.3611E-09	
t Critical one-tail	1.73406361	
P(T<=t) two-tail	6.7222E-09	
t Critical two-tail	2.10092204	

Table S3

Cellular CoQ_{10} concentration post CoQ_{10} supplementation

Control Cells (CoQ10 pmol/mg)	PABA Cells (CoQ10 pmol/mg)	Control/CoQ10 (CoQ10 pmol/mg)	PABA/CoQ10 (CoQ10 pmol/mg)
58.5	30	1064.81	1007
56.5	29	1689.05	1033
57.5	31.5	1010.06	1018
60	32	506.43	997
58	54	611.23	1029
60	31.5	973.07	986
61	29.5	1007.1	1040
59	27	798.67	993

	Control Cells (CoQ10 pmol/mg)	PABA Cells (CoQ10 pmol/mg)	Control/CoQ10 (CoQ10 pmol/mg)	PABA/CoQ10 (CoQ10 pmol/mg)
**Mean**	58.81	33.063	925.00	1012.88
**SD**	1.49	8.62	358.71	20.11
**SEM**	0.53	3.05	126.82	7.11

Comparison of groups >3 One-way Anova

Anova: Single Factor

SUMMARY

Groups	*Count*	*Sum*	*Average*	*Variance*
Control Cells pmol/mg	8	470.50	58.81	2.21
PABA Cells pmol/mg	8	264.50	33.06	74.25
Control/CoQ10	8	7660.42	957.55	128677.14
PABA/CoQ10	8	8103.00	1012.88	404.41

ANOVA

Source of Variation	*SS*	*df*	*MS*	*F*	*P-value*	*F crit*
Between Groups	7072813.56	3	2357604.52	73.01	2.3658E-13	2.95
Within Groups	904106.04	28	32289.50			

Total	7976919.60	31

TUKEY							
**Group 1**	**Group 2**	**Difference**	**n (Group 1)**	**n (Group 2)**	**SE**	**q**	**Studentized Range q Table**
Control Cells	PABA Cells	25.75	8	8	1.87	13.76	3.861
PABA Cells	Control/CoQ10	924.49	8	8	1.87	494.16	3.861
Control/CoQ10	PABA/CoQ10	55.32	8	8	1.87	29.57	3.861
PABA/CoQ10	Control Cells	954.06	8	8	1.87	509.97	3.861
Control Cells	Control/CoQ10	898.74	8	8	1.87	480.39	3.861
PABA/CoQ10	PABA Cells	979.81	8	8	1.87	523.73	3.861

Figure S4

Flow cytometry analysis of cellular LS fluorescence pre and post PABA treatment

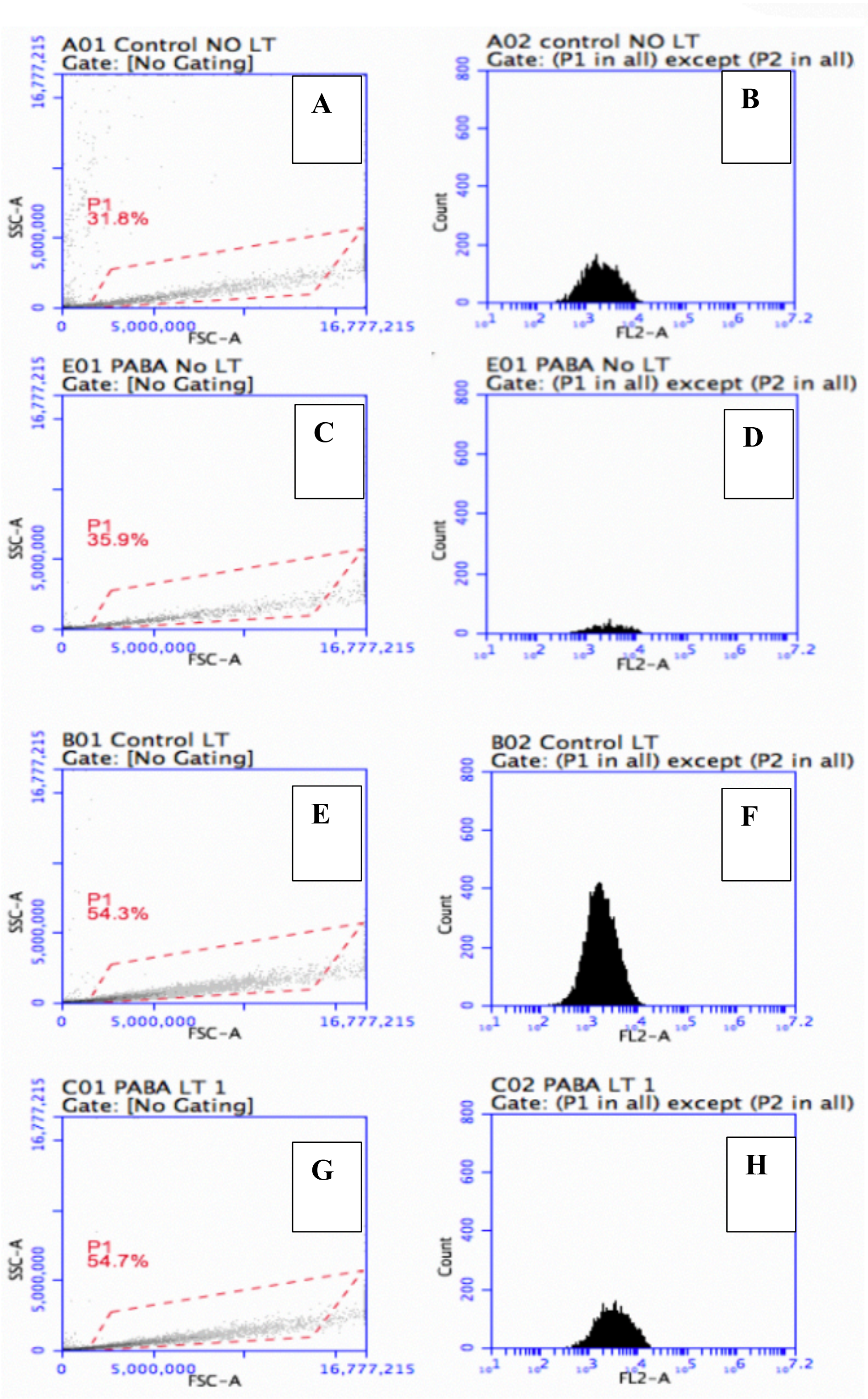

Demonstrates how the cells were gated on the flow cytometer. Images on the left show forward scatter compared to side scatter. Images on the right show the florescence value (AU) for each run. This also shows how the cells look without the LysoTracker (LT) probe. A/B – control cells with no LT and no PABA. C/D – cells with PABA no LT. E/F cells with LT no PABA. G/H cells with PABA and LT.

s

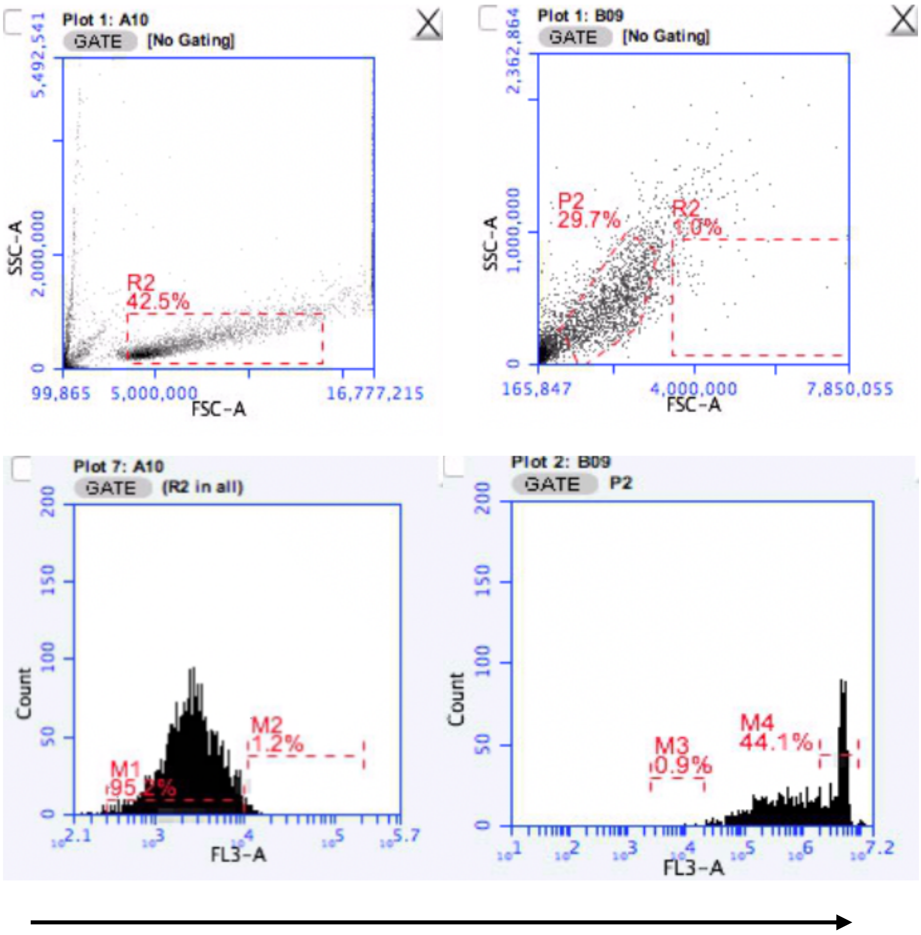

Demonstrates the shift in forward and side scatter between live and dead cells when cells treated with Propidium Iodide (PI) to asses cell distribution. Also shows the fluorescence shift in PI stained cells before and after death. R2/M1 represents live cells. P2/M4 represents Dead cells.

LIVE Cells Vs Dead Cells

	Control Cell median FL	PABA treated Cell median FL
	4,241.00	1,455.00
	3,167.00	1,361.00
	5,079.00	1,385.00
	5,847.50	1,369.00
	4,959.00	1,347.00
	5,402.00	1,329.00
	2,350.00	1,326.00
	2,677.50	1,344.00
	2,368.00	1,562.00
	2,696.00	1,553.00
	2,954.00	1,549.50
	2,894.00	1,573.00
	2,278.00	1,676.00
	2,220.00	1,580.00
	4,241.00	1,531.00
	3,167.00	1,603.00
	2,350.00	1,377.00
	2,677.50	1,410.00
	2,730.00	2,278.00
	2,870.00	2,220.00
	2,954.00	1,893.00
	2,894.00	1,773.00
	2,756.00	1,822.00
	4,689.00	1,999.50
	4,945.00	1,637.00
	4,809.00	1,410.00
**Mean**	3,469.83	1,590.88
**SD**	1134.78	265.59
**SEM**	222.55	52.08

t-Test: Two-Sample Assuming Equal Variances

	Variable 1	*Variable 2*
Mean	3469.82692	1590.88462
Variance	1287734.2	70542.2462
Observations	26	26
Pooled Variance	679138.223	
Hypothesized Mean Difference	0	
df	50	
t Stat	8.22064766	
P(T<=t) one-tail	3.7978E-11	
t Critical one-tail	1.67590503	
P(T<=t) two-tail	7.5956E-11	
t Critical two-tail	2.00855911	

<u>Table S5</u>

Mean LS fluorescence after standard pH solution exposure (AU)

pH	Mean exp 1	Mean exp 2	Mean exp 3	Mean exp4	Mean exp 5	Mean exp 6	Mean exp 7	Mean exp 8
**4.5**	19972.00	9967.00	8766.00	8978.00	8597.00	8307.00	8839.00	13136.00
**5.1**	8771.00	8604.00	9340.00	8969.00	10183.00	8967.00	9371.00	16776.00
**6**	8745.00	8982.00	9031.00	12127.00	10389.00	8811.00	8862.00	8060.00
**7**	9041.00	9081.00	8656.00	8631.00	8953.00	8796.00	8283.00	8177.00

pH Calibrant	Mean Fluorescence intensity across EXP (AU)
4.5	10820.25
5.1	10122.63
6	9375.88
7	8702.25

Table S6/7

Mean LS Fluorescence for controls and after PABA treatment (AU)

**Control**	9682	10639	9048	8858	9651	9530	11566	9643
**PABA**	9290	8523	9449	8505	8563	8538	9573	9138

**Control**	11465	9110	9574	9883	9608	8881	9939	11675
**PABA**	9801	9036	9419	9114	9041	9053	9180	9303

**Control**	9671	8630	9625	9909	8771	9813	9638	11472
**PABA**	9001	8892	8694	8640	9435	9512	8490	8754

**Control**	12138	11819	11480	11560	11342	11467	11341	11414
**PABA**	8814	8750	8760	9005	8848	9039	8133	8846

**Control**	9924	11321	11687	9476	11394	11137	11127	11223
**PABA**	9130	8835	8851	9167	9034	9169	9055	9302

**Control**	11345	11242	9907	12039	12885	11469	11515	9810
**PABA**	9446	9331	9349	9377	9308	9290	9312	9273

**Control**	11102	10004	9063	10906	11194	12271	12499	12519
**PABA**	9255	9311	9017	9241	9725	9113	9098	9519

**Control**	12633	11139	11649	11457	12205	9946	9519	9645
**PABA**	8815	8811	8775	9842	8883	9122	9205	8875

**Control**	11486	9452	9463	11307	9258	9358	9694	9468
**PABA**	8180	8882	8844	8163	8634	8087	8052	8288

**Control**	11290	9205	9227	9180	9443	12163	11001	9028
**PABA**	9395	9232	9237	9356	9324	9283	9322	9165

	Mean (AU)	SD (AU)	SEM (AU)
Control	10301.67	1464.47	151.049
PABA	9051.22	378.29	39.02

**Control**	9814	9343	1299	9902	9783	9157	9031	9925
**PABA**	9176	9190	9929	9096	9571	9212	9062	8992

**Control**	9002	9341	9093	9518	9606	9426
**PABA**	9034	9169	9055	9302	8701	8907

t-Test: Two-Sample Assuming Equal Variances

	Variable 1	*Variable 2*
Mean	10301.6702	9051.2234
Variance	2144674.09	143109.466
Observations	94	94
Pooled Variance	1143891.78	
Hypothesized Mean Difference	0	
df	186	
t Stat	8.01533506	
P(T<=t) one-tail	5.9021E-14	
t Critical one-tail	1.65308714	
P(T<=t) two-tail	1.1804E-13	
t Critical two-tail	1.97280011	

Mean LS Fluorescence after CoQ10 incubation (AU)

9793	9770	9744	10252	12272	9765	9712	10017
11782	9830	9788	9729	9162	9870	9826	9765
9823	9886	9699	9769	9939	9785	9791	9819
9316	9870	9779	9777	9844	9867	9722	9763

10129	10124	9991	18498	9896	9804	9927	9705
10123	10849	9737	10050	10208	10081	9847	10049
9780	9758	9737	9942	11874	9745	9713	9961
9854	9724	9752	9862	10055	10000	9976	9677
9275	9785	9757	9762	9291	9829	9782	9789
9942	9717	9743	9959	9776	9778	9859	9928
9808	9945	9757	10170	10108	9721	9764	10134
9851	9911	9775	9759	9845	9800		

Mean	SD	SEM
10002.91	984.8618	101.5807

Comparison of groups >3 One-Way Anova

ANOVA: Single Factor

SUMMARY

Groups	*Count*	*Sum*	*Average*	*Variance*
Control	94	968357	10301.67	2144674.09
PABA	94	850815	9051.22	143109.47
COQ	94	940274	10002.91	969952.81

ANOVA

Source of Variation	*SS*	*df*	*MS*	*F*	*P-value*	*F crit*
Between Groups	80169111.11	2	40084556	36.91	5.98407E-15	3.03
Within Groups	302969482.40	279	1085912			
Total	383138593.50	281				

TUKEY post hoc							
		Difference	n (Group 1)	n (Group 2)	SE	q	Studentized Range q Table
Control	PABA	1250.45	94	94	107.48	11.63	3.31

Control	CoQ	298.76	94	94	107.48	2.78	3.31
PABA	COQ	951.69	94	94	107.48	8.85	3.31
